# Inflammatory Potential of Four Different Phases of Calcium Pyrophosphate Relies on NF-κB Activation and MAPK Pathways

**DOI:** 10.3389/fimmu.2018.02248

**Published:** 2018-10-09

**Authors:** Laure Campillo-Gimenez, Félix Renaudin, Maud Jalabert, Pierre Gras, Marjolaine Gosset, Christian Rey, Stéphanie Sarda, Corinne Collet, Martine Cohen-Solal, Christèle Combes, Frédéric Lioté, Hang-Korng Ea

**Affiliations:** ^1^INSERM, UMR-S 1132, Université Paris Diderot (UFR Médecine), Sorbonne Paris Cité, Paris, France; ^2^CIRIMAT, Université de Toulouse, CNRS, INPT-ENSIACET, Toulouse, France; ^3^EA2496 Orofacial Pathologies, Imaging and Biotherapies, Dental School Faculty, Université Paris Descartes PRES Sorbonne Paris Cité, Montrouge, France; ^4^CIRIMAT, Université de Toulouse, CNRS, Université Paul Sabatier, Toulouse, France; ^5^Service de Biochimie, AP-HP, Hôpital Lariboisière, Paris, France; ^6^Service de Rhumatologie, AP-HP, Hôpital Lariboisière, Paris, France

**Keywords:** CPP crystals, microcrystal-induced arthritis, IL-1β, MAP kinase signaling, NF-κB pathway, macrophages, NLRP3 inflammasome

## Abstract

**Background:** Calcium pyrophosphate (CPP) microcrystal deposition is associated with wide clinical phenotypes, including acute and chronic arthritis, that are interleukin 1β (IL-1β)-driven. Two CPP microcrystals, namely monoclinic and triclinic CPP dihydrates (m- and t-CPPD), have been identified in human tissues in different proportions according to clinical features. m-CPP tetrahydrate beta (m-CPPTβ) and amorphous CPP (a-CPP) phases are considered as m- and t-CPPD crystal precursors *in vitro*.

**Objectives:** We aimed to decipher the inflammatory properties of the three crystalline phases and one amorphous CPP phase and the intracellular pathways involved.

**Methods:** The four synthesized CPP phases and monosodium urate crystals (MSU, as a control) were used *in vitro* to stimulate the human monocytic leukemia THP-1 cell line or bone marrow-derived macrophages (BMDM) isolated from WT or NLRP3 KO mice. The gene expression of pro- and anti-inflammatory cytokines was evaluated by quantitative PCR; IL-1β, IL-6 and IL-8 production by ELISA; and mitogen-activated protein kinase (MAPK) activation by immunoblot analysis. NF-κB activation was determined in THP-1 cells containing a reporter plasmid. *In vivo*, the inflammatory potential of CPP phases was assessed with the murine air pouch model via cell analysis and production of IL-1β and CXCL1 in the exudate. The role of NF-κB was determined by a pharmacological approach, both *in vivo* and *in vitro*.

**Results:** *In vitro*, IL-1β production induced by m- and t-CPPD and m-CPPTβ crystals was NLRP3 inflammasome dependent. m-CPPD crystals were the most inflammatory by inducing a faster and higher production and gene expression of IL-1β, IL-6, and IL-8 than t-CPPD, m-CPPTβ and MSU crystals. The a-CPP phase did not show an inflammatory property. Accordingly, m-CPPD crystals led to stronger activation of NF-κB, p38, extracellular signal-regulated kinase 1/2 (ERK1/2) and c-Jun N-terminal kinase (JNK) MAPKs. Inhibition of NF-κB completely abrogated IL-1β and IL-8 synthesis and secretion induced by all CPP crystals. Also, inhibition of JNK and ERK1/2 MAPKs decreased both IL-1β secretion and NF-κB activation induced by CPP crystals. *In vivo*, IL-1β and CXCL1 production and neutrophil infiltration induced by m-CPPD crystals were greatly decreased by NF-κB inhibitor treatment.

**Conclusion:** Our results suggest that the inflammatory potential of different CPP crystals relies on their ability to activate the MAPK-dependent NF-κB pathway. Studies are ongoing to investigate the underlying mechanisms.

## Introduction

Calcium pyrophosphate dihydrate (CPPD) microcrystal deposition disease affects more than 17.5% of people over 75 years old (1, 2). Monoclinic and triclinic CPPD phases (m-CPPD and t-CPPD, respectively) are the two types of CPPD crystals identified in synovial fluids at the time of acute arthritis, but the existence of precursor phases such as m-CPP tetrahydrate beta (m-CPPTβ) and amorphous CPP (a-CPP) have been suggested *in vitro* (3, 4). CPPD microcrystals are responsible for numerous clinical manifestations including asymptomatic CPPD, osteoarthritis with CPPD, and acute and chronic CPP crystal-induced arthritis, involving from one to several joints (5). A few studies suggest that differences in the size, structure, surface area, shape of crystals and proportion of the different crystals may explain these wide clinical phenotypes. For example, smaller crystals are more active than larger ones: the crystal surface plays a major role in determining cellular response and m-CPPD may be more active than t-CPPD (6–10). In line with these pre-clinical results, acute inflammatory synovial fluids contain a greater proportion of m-CPPD crystals and low inflammatory samples contain a greater proportion of t-CPPD crystals (7).

Like monosodium urate (MSU) and basic calcium phosphate (BCP) microcrystals, m- and t-CPPD microcrystals are responsible for recurrent inflammatory flares by their ability to induce production of a large panel of pro-inflammatory mediators such as interleukin-1β (IL-1β), IL-6, IL-8, or tumor necrosis factor-α (TNF-α) (11–13). The *in vivo* inflammatory response induced by microcrystals is clearly reported in mouse intra-articular, intraperitoneal or air pouch models of crystal injection. Using IL-1 receptor knock-out (KO) mice, mice treated with IL-1RA (IL-1 receptor antagonist) or depleted-macrophage mice, those studies demonstrated that acute inflammatory phase is mainly orchestrated by IL-1β secreted by resident macrophages after crystal detection (12, 14, 15). IL-1β production occurs through a 2-step process involving (i) nuclear factor-κB (NF-κB) activation that leads to pro-IL1β formation and (ii) NLRP3 (NOD-like Receptor family, Pyrin domain containing 3) inflammasome activation that permits caspase-1 to cleave pro-IL-1β into mature IL-1β (16). IL-1β, as well as crystals themselves, are then able to activate the production of multiple pro-inflammatory cytokines by macrophages, endothelial cells, fibroblasts, synoviocytes. Especially, IL-8 production is responsible for a massive polymorphonuclear neutrophil (PMN) recruitment in the site of crystal injection which participates to the amplification of inflammatory response (17).

Microcrystals activate cellular responses after a direct crystal-cell membrane contact or a specific crystal-cell receptor interaction, either through naked crystals or via proteins adsorbed on the crystal surface (13, 18–21). As reported, naked MSU, BCP and CPPD crystals stimulate cellular receptors such as integrin CD11b/CD18, the Fc receptor CD16, the cell surface-expressed immunoglobulin triggering receptor expressed on myeloid cell 1 and Toll-like receptors (TLR) 2 and 4 (13, 22–25). The interaction of crystals with cell membrane and/or cell receptors stimulates numerous intracellular signaling pathways including NLRP3 inflammasome complex, intracellular calcium mobilization, Src and Syk kinases, myeloid differentiation factor 88 (MyD88)-dependent downstream signaling as well as extracellular signal-regulated kinase 1/2 (ERK1/2), p38 and c-Jun N-terminal kinase (JNK) mitogen-activated protein kinases (MAPKs) (11, 12, 18, 26–29). These intracellular pathways activate transcription factors such as activator protein 1 and NF-κB, which regulate inflammatory gene expression (19, 28).

A few studies have shown that m-CPPD microcrystals are more inflammatory than t-CPPD microcrystals, inducing greater and longer PMN recruitment and greater production of prostaglandin-E2, reactive oxygen species and TNF-α; however, the underlying mechanisms and cellular signaling pathways have not been investigated (9, 10). Similarly, no study has yet assessed the potential of each CPPD crystal to induce the production of IL-1β, the main cytokine orchestrating the crystal-induced inflammatory reaction. Furthermore, whether precursors of CPPD crystals such as m-CPPTβ and a-CPP phases can stimulate inflammatory mediators and cellular responses is unknown. Thus, we aimed to precisely characterize the inflammatory potential of these four pure phases of CPP by studying synthesis signaling of pro-inflammatory cytokines, in particular the production of pro- and mature IL-1β, and the MAPK/NF-κB activation pathway.

## Materials and methods

### *In vitro* crystal synthesis and characterization

MSU crystals were obtained by spontaneous precipitation of uric acid in NaOH solution (0.01 M) at 60°C as described (30). Four different CPP pure phases were synthesized and characterized as previously reported (4). These compounds were characterized by X-ray diffraction (Seifert XRD-3000TT diffractometer with Cu Kα radiation, in the 2θ range 2°-70° with step size 0.02° and scan step time 16 s at 298 K), FTIR spectroscopy (Thermo Nicolet 5700 Fourier-transform infrared spectrometer, range 4,000–400 cm^−1^, 64 scans, 4 cm^−1^ resolution, transmission mode with powder samples in KBr pellets) and scanning electron microscopy (SEM, Leo 435 VP microscope, accelerating voltage: 7 kV, samples were silver-plated before observation). Chemical analysis of pyrophosphate and calcium was performed as follows: standard spectrophotometric (Hitachi U-1100 spectrometer, λ = 460 nm) determination of the yellow phosphovanadomolybdic acid complex was used to determine phosphate concentration after hydrolysis of the pyrophosphate (at 100°C in acidic medium during 1 h) into phosphate ions; calcium concentration was determined by complexometry with ethylene diamine tetra-acetic acid (EDTA). The specific surface area of the sample was evaluated on a Quanta chrome Instruments Monosorb Nova 1,000 with the Brunauer–Emmett–Teller method (nitrogen adsorption). Crystals/particles were dispersed by brief sonication and suspended at 2 mg/ml in phosphate buffered saline (PBS). They were prepared under endotoxin-free conditions and tested negative with Pierce *Limulus amebocyte* Assay (Thermo Fisher Scientific).

Thorough physicochemical characterization of the four synthesized CPP samples showed that each consisted of a pure phase: three were pure crystalline phases (m-CPPD, t-CPPD, and m-CPPTβ) and one was an amorphous phase (a-CPP) (4).

### Mice and cells

Eight-week-old C57Bl/6J mice were used for *in vivo* or *in vitro* experiments. Wild-type (wt) mice were purchased from Janvier Lab (Le Genest-St-Isle, France). NLRP3 KO (*nlrp3*^−/−^) mice were gifted by Dr. JL Connat (INSERM 866, Université Bourgogne Franche-Comté, Dijon, France). Mice were maintained in cages (max. 6/cage) in a facility with 12 h light/dark cycles. Mice were fed diets *ad libitum*.

For *in vitro* experiments, wt and *nlrp3*^−/−^ bone marrow cells were recovered from tibia and femoral bones of mice and seeded in 24-well plates at 2 × 10^6^ cells/ml in L929-conditioned RPMI 1640 media as described (31). Every 2 days, cells were washed and the media renewed until complete differentiation of bone marrow-derived macrophages (BMDMs). BMDMs were maintained in RPMI 1640 supplemented with 10% fetal bovine serum (FBS), HEPES (25 mM), L-glutamine (2 mM), penicillin (100 U/ml), and streptomycin (100 μg/ml) and were primed overnight with ultrapure lipopolysaccharide (LPS; 20 ng/ml).

Human monocytic leukemia (THP-1) cells were maintained in the same complete RPMI 1640 media and were primed for 6 h with phorbol 12-myristate 13-acetate (PMA, 0.5 μM), washed with PBS1X, then plated in 24-well dishes at 3 × 10^5^ cells/well and left overnight in complete media. THP-1 cells containing an NF-κB-inducible Luc reporter construct (THP-1-Lucia™ NF-κB Cells, Invivogen) were used to assess NF-κB activation. THP-1-Lucia cells were maintained in the same complete RPMI 1640 media supplemented with pyruvate 1 mM (Gibco) and the selection antibiotic zeocin (Invivogen).

Primed BMDMs and THP-1 cells were washed twice with PBS and stimulated at the indicated times with MSU or CPP crystals (200 μg/ml) in FBS-free media. For some experiments, THP-1 cells were incubated with inhibitors of NF-κB (BAY-11-7085, 10 μM), p38 (SB203580, 10 μM), JNK (SP600125, 10 μM), or p42/44 (PD98059, 100 μM) (all Tocris, R&D Systems) 30 min before crystal stimulation. After crystal stimulation, supernatants were collected for cytokine quantification and cells were lysed for mRNA quantification or protein expression analysis.

THP-1-Lucia cells were washed twice and stimulated for 8 h with MSU or CPP crystals (200 μg/mL) or heat-killed *Listeria monocytogenes* (HKLM, Invivogen: positive control of NF-κB induction) in FBS-free medium. Absolute luciferase activity was determined in the supernatants with a Xenius luminometer (SAFAS Monaco) according to the manufacturer's instructions. Relative luminescence units were normalized by the proportion of live cells determined with the Pierce LDH assay kit (Thermo Fisher Scientific), then the NF-κB reporter relative activity was calculated.

### Mouse air pouch model

Eight-week-old wild type C57Bl/6J mice were used for *in vivo* experiments. After purchase, mice were housed 1 week before experimentation. Air pouches were created by two dorsal subcutaneous injections of 3 ml sterile air (day 0 and day 3), under isoflurane anesthesia. At day 6, PBS or crystals (1 mg/ml, diluted in PBS) were injected directly into the air pouch. In case of NF-κB study, PBS or BAY-11-7085 (NF-κB inhibitor, 200 μg/mice) were injected directly into the air pouches 30 min before PBS or crystal injection. At 6 and 24 h post-crystal injection, air pouches were washed twice with 2 ml of PBS under ketamine/xylazine anesthesia then mice were sacrificed by cervical dislocation. Mice displayed normal behavior after air pouch creation and crystal injection. No painkiller was necessary. The air pouch lavages were used for cytokine measurement and cell counting/staining.

### Cytokine quantification

Cytokine production in supernatant or air pouch lavage was measured by using ELISA kits [IL-1β, IL-8 (Invitrogen), CXCL1 (R&D System), IL-6 (Roche Diagnostics)].

### mRNA quantification

Primed THP-1 cells were lysed with TRizol reagent (Invitrogen), 6 h after crystal stimulation, and total RNA was extracted by using the ISOLATE II RNA kit (Bioline). First, 500 ng of total RNA were reverse transcript to cDNA using the High Capacity cDNA Reverse Transcription Kit (Applied Biosystem) (LifeECO Thermal Cycler, Bioer Technology). Then, real time quantitative PCR was performed with 25 ng of cDNA using the SensiFAST SYBR No-ROX Kit (Bioline) for 40 cycles (95°C for 5 s, 60°C for 30 s) (LightCycler®480 Instrument, Roche). Sequences of primers for real time qPCR are listed in Table 1.

**Table 1:** Human primers used for gene expression analysis.

		**Forward**	**Reverse**
Human	IL-1β	TTCGAGGCACAAGGCACAA	TGGCTGCTTCAGACACTTGAG
	IL-8	GAGCCAGGAAGAAACCACCG	TGGCAAAACTGCACCTTCACA
	IL-6	GGCACTGGCAGAAAACAACC	GCAAGTCTCCTCATTGAATCC
	TNF-α	CCCATGTTGTAGCAAACCCTC	TATCTCTCAGCTCCACGCCA
	NLRP3	CTTCCTTTCCAGTTTGCTGC	TCTCGCAGTCCACTTCCTTT
	ASC	AGTTTCACACCAGCCTGGAA	TTTTCAAGCTGGCTTTTCGT
	Caspase-1	AGCCACATCGCCAGACAC	GCCCAATACGACCAAATCC
	IL-1RA	GGAAGATGTGCCTGTCCTGT	CCTTCGTCAGGCATATTGGT
	IL-10	AAGACCCAGACATCAAGGCG	AATCGATGACAGCGCCGTAG
	IL-37	CGATTCTCCTGGGGGTCTCT	GCTTCATCAGTTTCTCCTTCTTCA
	TGF-β	GGTGGAAACCCACAACGAAAT	GAGCAACACGGGTTCAGGTA
	GAPDH	AGCCACATCGCCAGACAC	GCCCAATACGACCAAATCC

*All primers were designed by using PrimerBLAST*.

### Flow cytometry

A total of 1 × 10^5^ cells from air pouch lavages was stained with anti-F4/80-APC and anti-Ly6G-PE mouse monoclonal antibodies (mAbs; Miltenyi Biotechnology) for 20 min at room temperature, washed in PBS and analyzed with the BD FACS Canto II cytometer (BD Bioscience). Data were analyzed with BDFACS Diva software (BD Bioscience).

### Western blot analysis

Cell lysates were obtained from primed THP-1 cells or BMDM after crystal stimulation. Cells were washed with ice-cold PBS and lysed for 30 min on ice with Triton X-100 based-buffer plus phosphatase (orthovanadate, Sigma-Aldrich) and protease inhibitor cocktail (Sigma-Aldrich). Cell lysates were collected after centrifugation (7,500 g, 15 min) and total protein concentration was determined with the BCA Pierce Assay (Thermo Fisher Scientific). An amount of 30 μg protein was used for SDS-PAGE separation and transferred to PVDF membranes for western blot analysis. Anti-phosphorylated (P-) or total p38, p42/44 (ERK1/2) and JNK rabbit mAbs (1/1,000), as well anti-NLRP3 (1/500) rabbit mAbs were purchased from Cell Signaling. Anti-tubulin pAb was from Santa Cruz Biotechnology (1/1,000), anti-actin pAb was from Sigma-Aldrich (1/5,000).

### Statistical analysis

Data are reported as mean ± SEM. After verification of Gaussian distribution and homogenous variance of each group, two-way ANOVA test or multiple *t*-test followed by false discovery rate (FDR) correction were used to compare experimental conditions. Otherwise, Kruskal-Wallis test with FDR correction was chosen. For parametric or non-parametric tests, linear correlation was determined by the non-parametric Spearman test. The significance level was set at *P* < 0.05. GraphPad Prism 7.0 (San Diego, CA) was used for analysis.

## Results

### Physicochemical and morphological characteristics of CPP phases

**Figure 1:**
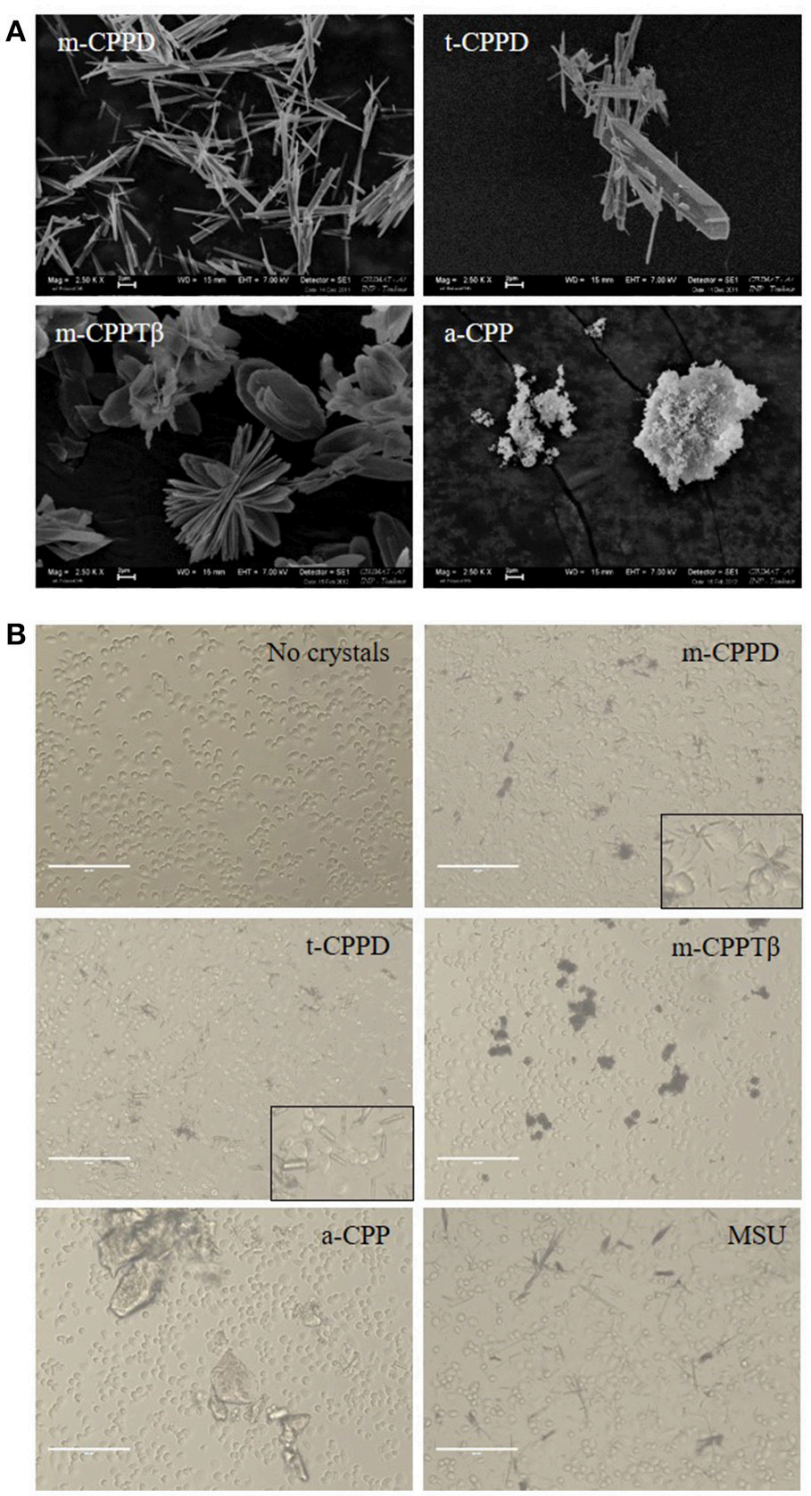
Physicochemical and morphological characteristics of the four CPP phases. **(A)** SEM micrographs of the four synthetic pure CPP phases tested in this study (scale bar = 2 μm). **(B)** Optical micrographs of THP-1/crystal culture by EVOS system microscopy (X20).

The physicochemical characterization of each CPP phase is summarized in Table 2. Electron microscopy of each pure synthetic CPP phases allowed for characterizing the different morphologies of crystals or particles (Figure 1A). As observed for biological specimens of CPPD crystals, the synthetic crystals of the CPPD phases were acicular crystals, some arranged as bundles, with large heterogeneity in size, and t-CPPD crystals larger than m-CPPD crystals. Measured sizes were from 1 to 30 μm for t-CPPD, with maximum width 5 μm, and 1–25 μm for m-CPPD, with maximum width 1 μm. m-CPPTβ had a faceted plate morphology, with diameter up to 15 μm. The a-CPP phase appeared as round microparticles consisting of agglomerated smaller particles of about 100 nm. On optical microscopy of THP-1 cells cultured with CPP phases, m-CPPD crystals exhibited a needle-shaped structure and were the smallest and were more dispersed in cell culture than the others, whereas t-CPPD crystals had a rectangular shape, m-CPPTβ crystals were platelet-like and formed aggregates and the a-CPP phase corresponded to large shapeless particles (Figure 1B).

**Table 2:** Physicochemical characteristics of the four CPP phases.

	**Size, length × width (μm)**	**Specific surface area (m^2^/g)**	**Morphology**	**Aggregates formed in culture**	**Ca/P atomic ratio**
m-CPPD	1–25 × 1	5.0 ± 0.2	Thin needles	–	1.01 ± 0.02
t-CPPD	1–30 × 5	1.3 ± 0.1	Thick needles	+	1.01± 0.02
m-CPPTβ	12–15 × 4	4.8 ± 0.2	Platelets	+++	1.00 ± 0.02
a-CPP	0.1	25.7 ± 0.5	Round shape aggregates of small particles	++	1.00 ± 0.02

*m- and t-CPPD, monoclinic and triclinic calcium pyrophosphate dihydrate; m-CPPTβ, monoclinic calcium pyrophosphate tetrahydrate β; a-CPP, amorphous CPP; Ca/P, calcium on phosphorus molar ratio of the sample*.

### Monoclinic CPPD microcrystals are the most inflammatory CPP microcrystals

We first assessed the inflammatory potential of different phases of CPP crystals *in vitro*. In contrast of other pro-inflammatory cytokines that required only a priming signal, IL-1β production induced by microcrystals depends on 2 signals: first, a priming signal through activation of innate immunity receptors such as TLRs induces the synthesis and accumulation of proIL-1β, the precursor form of the cytokine and second, a maturation signal through NLRP3 inflammasome activation leads to IL-1β secretion. Therefore, THP-1 and BMDM are primed with PMA and LPS, respectively, to induce their activation and the accumulation of proIL-1β, then stimulated with the four pure and pyrogen-free synthetic CPP phases: m-CPPD, t-CPPD, m-CPPTβ, and a-CPP phases (32). The effect of CPP crystals was also compared to that of MSU crystals.

**Figure 2:**
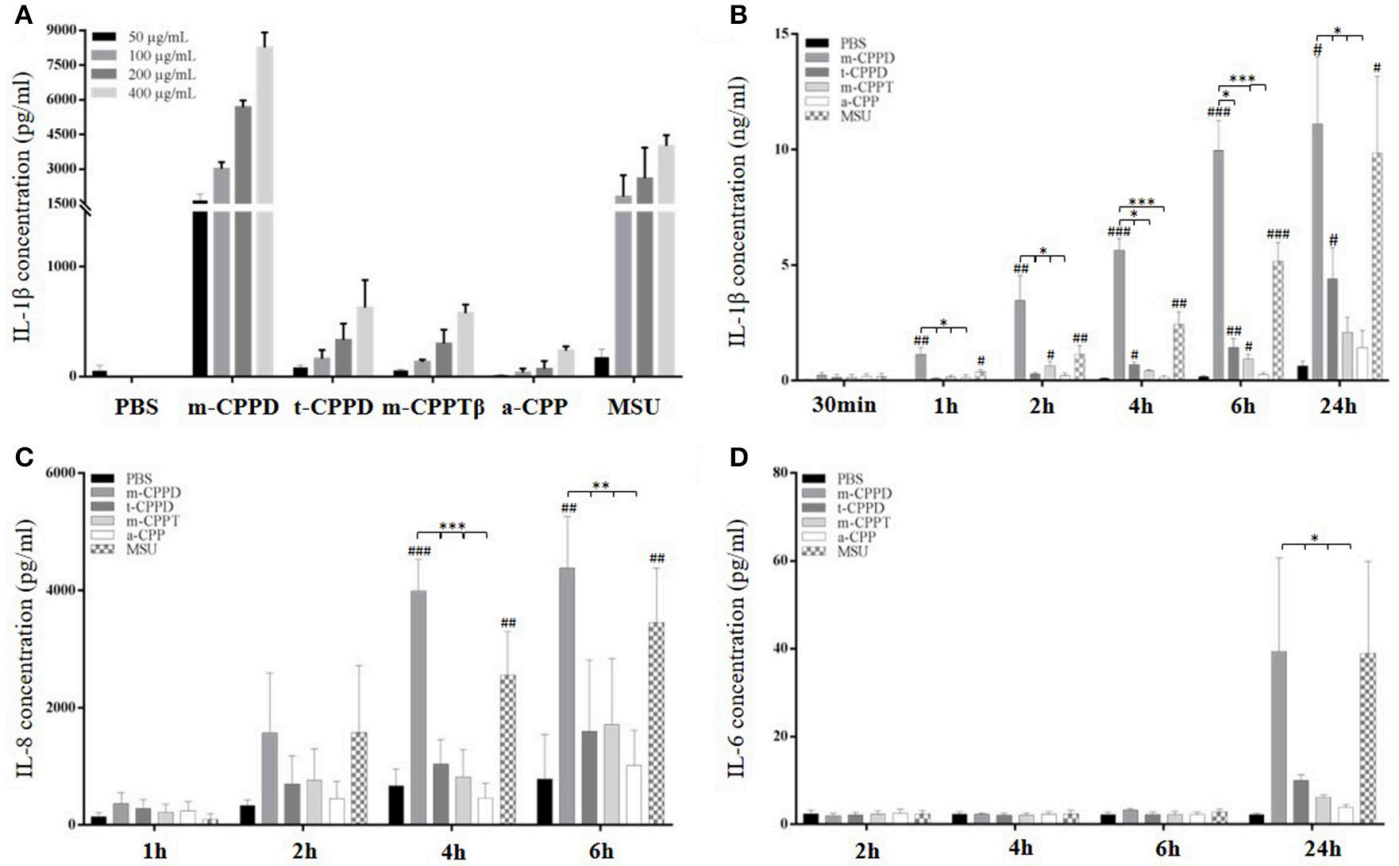
Kinetics of IL-1β, IL-8, and IL-6 production in response to CPP phases. THP-1 cells were primed the day before experiments. Cells were harvested at different times of crystal stimulation as indicated. **(A)** IL-1β concentration was assessed in supernatant after 6-h stimulation with different doses of crystals. **(B–D)** Cells were stimulated with 200 μg/ml of different crystals and, at the indicated time, supernatants were collected to quantify IL-1β **(B)**, IL-8 **(C)**, and IL-6 **(D)** concentrations (*n* = 3). Kruskal-Wallis test with FDR correction between crystal and PBS (#) or between CPP crystals (*). ^#^,*p < 0.05; ^*##*^,**p < 0.01; ^*###*^,***p < 0.001.

**Figure 3:**
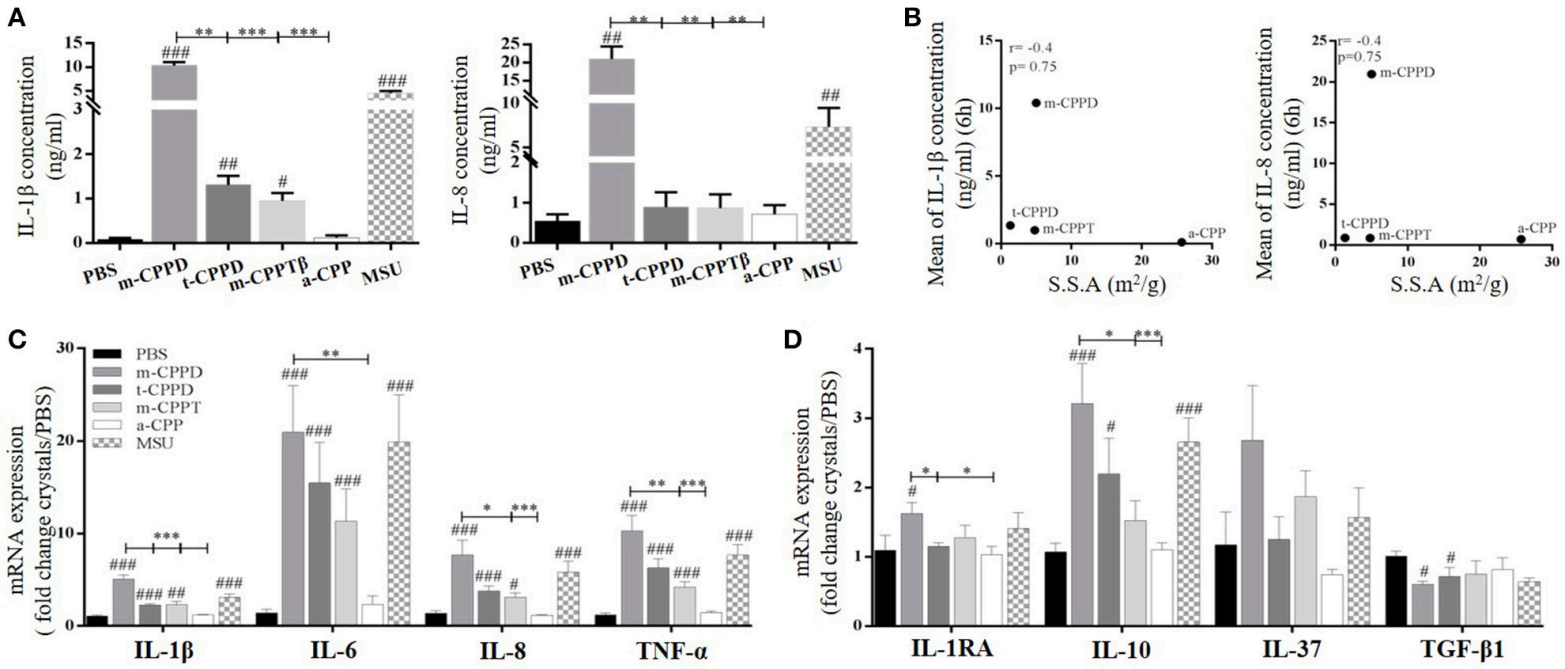
Differential effects of CPP phases on the inflammatory response *in vitro*. THP-1 were primed the day before experiments. After 6 h of crystal stimulation, supernatants, and total RNA were collected. **(A)** IL-1β (*n* = 10) and IL-8 (*n* = 3) concentrations were quantified from cell supernatants by ELISA. Kruskal-Wallis test with FDR correction between crystals and PBS (#) or between CPP crystals (*); **(B)** Correlation analysis between mean of IL-1β/IL-8 concentrations measured at 6 h after crystal stimulation and specific surface area (S.S.A) of each CPP phase. Spearman *t*-test. **(C,D)** THP-1 cells were lysed for quantification of IL-1β, IL-6, IL-8, and TNF-α (*n* = 10) and IL-1RA, IL-10, IL-37, and TGF-β (*n* = 3) mRNA expression by qPCR. Kruskal-Wallis test with FDR correction between crystal and PBS (#) or between CPP phases (*). ^#^,*p < 0.05; ^*##*^,**p < 0.01; ^*###*^,***p < 0.001.

As compared with control stimulation, the amount of IL-1β in cell culture medium increased with time after MSU, m-CPPD, t-CPPD, and m-CPPTβ crystal stimulation; only the a-CPP phase did not induce IL-1β secretion. IL-1β production was dependent on crystal dose, 50 μg/ml of m-CPPD crystals induced high amount of IL-1β secretion while the same concentration of t-CPPD and m-CPPTβ had no effect on IL-1β production after 6 h stimulation (Figure 2A). m-CPPD microcrystals rapidly induced IL-1β production, which significantly increased after 1 h of crystal stimulation, whereas the effect of t-CPPD and m-CPPTβ was delayed (Figure 2B). Similarly, IL-8 production induced by m-CPPD microcrystals began earlier than with the other microcrystals (Figure 2C). In contrast, IL-6 production occurred later and was evidenced only at 24 h of stimulation (Figure 2D). At peak production, that is, 6 h after stimulation, and for a same dose of crystals (200 μg/ml), the amount of IL-1β and IL-8 induced by m-CPPD crystals was greater than that induced by t-CPPD, m-CPPTβ, or MSU crystals (Figure 3A). The differential inflammatory property of CPP microcrystals based on IL-1β/IL-8 production by THP-1 cells was not correlated with the values of the specific surface area (SSA) of the studied CPP phases (Figure 3B).

Moreover, m-CPPD crystals had a higher capacity to induce the expression of inflammatory cytokine genes (IL-1β, IL-6, IL-8, TNF-α) on primed THP-1 cells, compared to t-CPPD and m-CPPTβ crystals, but the a-CPP phase did not induce gene expression (Figure 3C). The pattern of production of IL-1β and IL-8 was strongly associated with their gene expression profile. CPP microcrystals also differentially modulated the gene expression of anti-inflammatory cytokines. IL-1RA and IL-10 mRNA expressions were higher at 6 h after stimulation with m-CPPD crystals than t-CPPD and m-CPPTβ microcrystals; Transforming growth factor-β1 (TGF-β1) expression was slightly decreased only after m-CPPD crystal stimulation and IL-37 expression was unchanged under all types of CPP crystal stimulation (Figure 3D).

**Figure 4:**
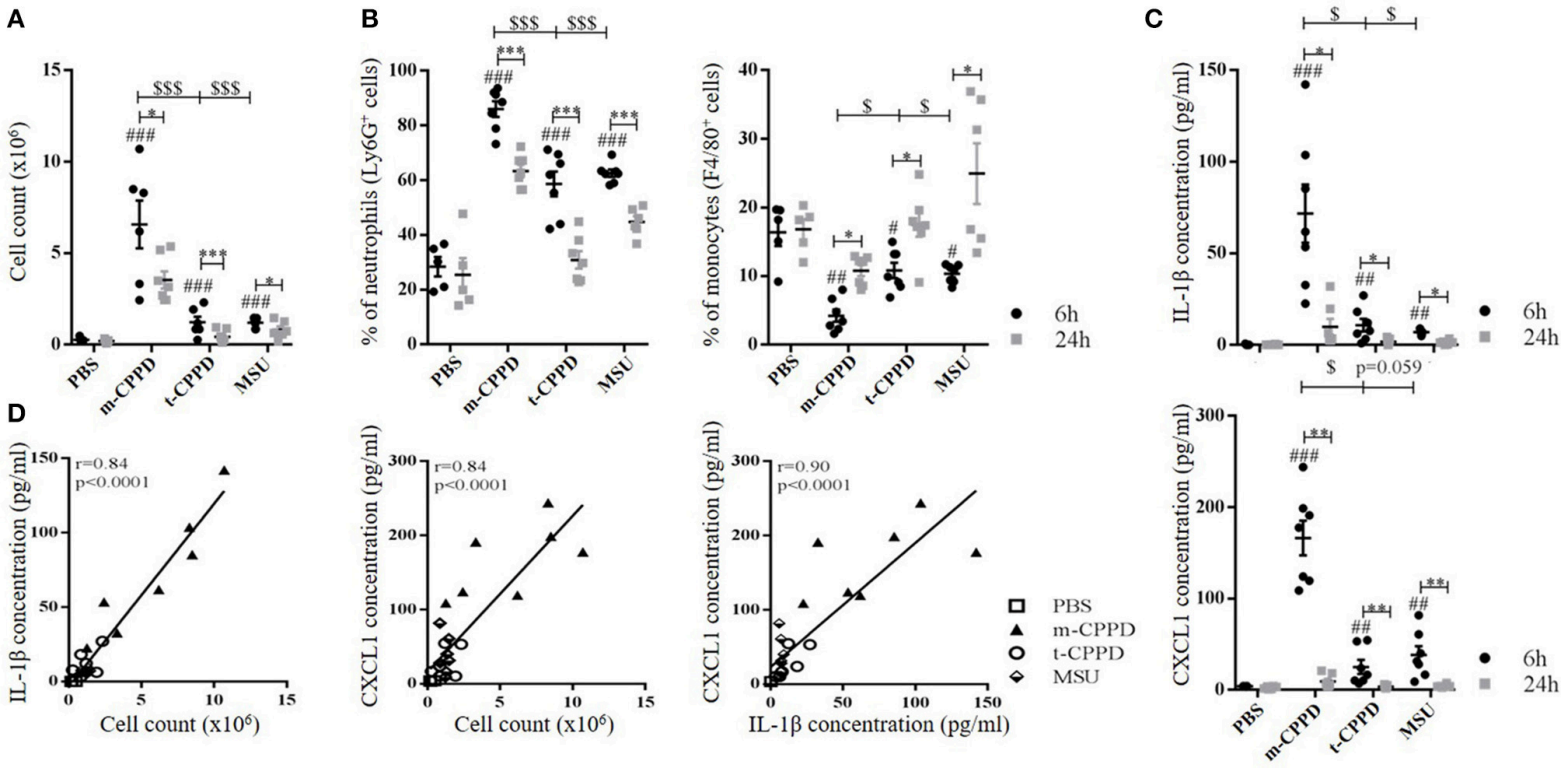
CPP crystals differentially trigger inflammatory response *in vivo*. Air pouches were created by two injections of 3 ml of sterile air at day 0 and day 3. At day 6, crystals or PBS were injected into the air pouches and inflammation was assessed 6 and 24 h after. **(A–C)** From the air pouch lavage, number of infiltrated cells was evaluated by cell counting with Trypan blue exclusion **(A)**; proportion of recruited neutrophils (PMN) and macrophages among total cells was determined by flow cytometry after staining with anti-Ly6G-PE and F4/80-APC monoclonal antibodies (mAbs), respectively **(B)**; IL-1β and CXCL1 concentrations were quantified by ELISA **(C)**. Two-way ANOVA with FDR correction between crystal and PBS at 6 h (#) or m-CPPD and other crystals ($) or between 6 and 24 h (*). ^*#, $*^,*p < 0.05; ^*##, $$*^,**p < 0.01; ^*###, $$$*^,***p < 0.001. **(D)** Correlation analysis between cell count and IL-1β (Left) or CXCL1 level (Middle) or between IL-1β and CXCL1 levels (Right), at 6 h of crystal stimulation. Spearman test, *p < 0.05; **p < 0.01; ***p < 0.001.

To confirm the different inflammatory properties, we compared *in vivo* inflammation induced by m-CPPD and t-CPPD microcrystals, the only two phases identified in human synovial fluid, by using the murine air pouch model. We assessed the kinetics of the inflammatory response at 6 and 24 h after MSU, m-CPPD and t-CPPD crystal injection (Figure 4). All three microcrystals conferred an acute inflammatory reaction within 6 h, characterized by an increase in cell number in the air pouch lavage (Figure 4A) corresponding to high PMN recruitment (Figure 4B) and an increase in IL-1β and CXCL1 production (Figure 4C). CXCL1 level in the air pouch was positively correlated with IL-1β level and cell infiltrates with IL-1β and CXCL1 levels (Figure 4D).

At 24 h, according to the decrease in total cell number, PMN infiltration induced by microcrystal injection was significantly decreased (m-CPPD 6 h: 86 ± 9%, 24 h: 63 ± 2%; t-CPPD 6 h: 59 ± 4%, 24 h: 31 ± 3%; MSU 6 h: 63 ± 1%, 24 h: 45 ± 2%; two-way ANOVA: *p* < 0.001), which suggests the initiation of inflammation resolution. This finding was favored by the substantial decrease in IL-1β production and the abolition of CXCL1 production. The inflammation secondary to m-CPPD microcrystals was higher and lasted longer than that induced by t-CPPD and MSU microcrystals (Figure 4).

Taken together, these results confirmed that the four CPP phases possessed different inflammatory properties, with m-CPPD crystals being the most inflammatory, followed by t-CPPD and m-CPPTβ, which had the same inflammatory potential. By contrast, the a-CPP phase was not inflammatory. All *in vitro* experiments of cell stimulation by CPP phases have been performed in FBS-free media. Therefore, the differential inflammatory potential of the 4 CPP phases cannot be explained by crystal coating.

### CPP crystal-induced IL-1β production depends on NLRP3 inflammasome activation

**Figure 5:**
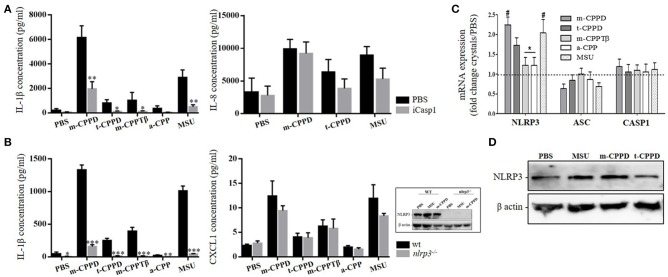
CPP crystal-induced IL-1β production is NLRP3-dependent. **(A)** Human IL-1β concentration from PMA-primed THP-1 cell cultures after 6 h stimulation by different crystals in absence (PBS) or presence of caspase 1 inhibitor (iCasp1, 10 μM, *n* = 5). Two-way ANOVA test with FDR correlation between PBS and iCasp1. **(B)** Mouse IL-1β and CXCL-1 concentrations from wild type (wt, Left) and *nlrp3*^−/−^ (Right) BMDM cultures (*n* = 6) after 6 h stimulation by different crystals. 2-way ANOVA test with FDR correlation between crystal and PBS (#) or between CPP crystals (*). ^#^,*p < 0.05; ^*##*^,**p < 0.01; ^*###*^,***p < 0.001. **(C)** mRNA quantification of NLRP3, ASC and pro-Casp1 gene expression from PMA-primed THP-1 cells stimulated by different crystals. Kruskal-Wallis test with FDR correlation between crystals and PBS (#) or between CPP crystals (*). ^#^,*p < 0.05; ^*##*^,**p < 0.01; ^*###*^,***p < 0.001. **(D)** Immunoblot analysis of NLRP3 protein expression from primed THP-1 cells or BMDM isolated from wt or nlrp3^−/−^ mice (box in **B**) after 6 h of crystal stimulation; one representative of 3 independent experiments.

NLRP3 inflammasome regulates IL-1β production induced by MSU, BCP or a mixed of CPPD crystals (12, 15). Whether IL-1β secretion induced by each pure phase of CPP crystals is also NLRP3-dependent is unknown. Using a pharmacological approach, we first observed that IL-1β maturation by different CPP phases was secondary to caspase 1 (Casp1) activity. Thus, CPP crystal-stimulated IL-1β production was drastically decreased in primed THP-1 cells treated with a Casp1 inhibitor (iCasp1) (Figure 5A, left). As expected, Casp1 inhibition did not alter CPP crystal-induced IL-8 production (Figure 5A, right). Then, we confirmed the role of NLRP3 inflammasome in NLRP3 knock-out model. We observed that CPP crystal-induced IL-1β production was abrogated in primed *nlrp3*^−/−^ BMDM (Figure 5B, left). Similarly, CPP crystal-induced IL-8 production was unchanged in *nlrp3*^−/−^ BMDM compared to wt BMDM (Figure 5B, right). Next, we assessed whether CPP crystals modulated the expression of NLPR3 and its complex-forming partners, namely the adaptor protein ASC (*Apoptosis associated Speck-like protein containing a Caspase recruitment domain*) and Casp1. CPP crystals did not induce the gene expression of ASC and pro-Casp1 but did differentially increase both the gene and protein expression of NLRP3 (Figures 5C,D). Accordingly, the effect of m-CPPD crystals was more important than t-CPPD and m-CPPTβ crystals. And interestingly, the differential effect on NLRP3 gene and protein expression was mild compared to the differential effect observed for IL-1β, IL-8, IL-6, or TNF-α production. In first conclusion, these results suggest that the inflammatory potential of different CPP crystals is mediated by their ability to differentially (i) amplify the priming signal increasing the expression of multiple inflammatory cytokines, and (ii) activate the NLRP3 inflammasome leading to IL-1β production.

### NF-κB regulates CPPD microcrystal-induced inflammation

**Figure 6:**
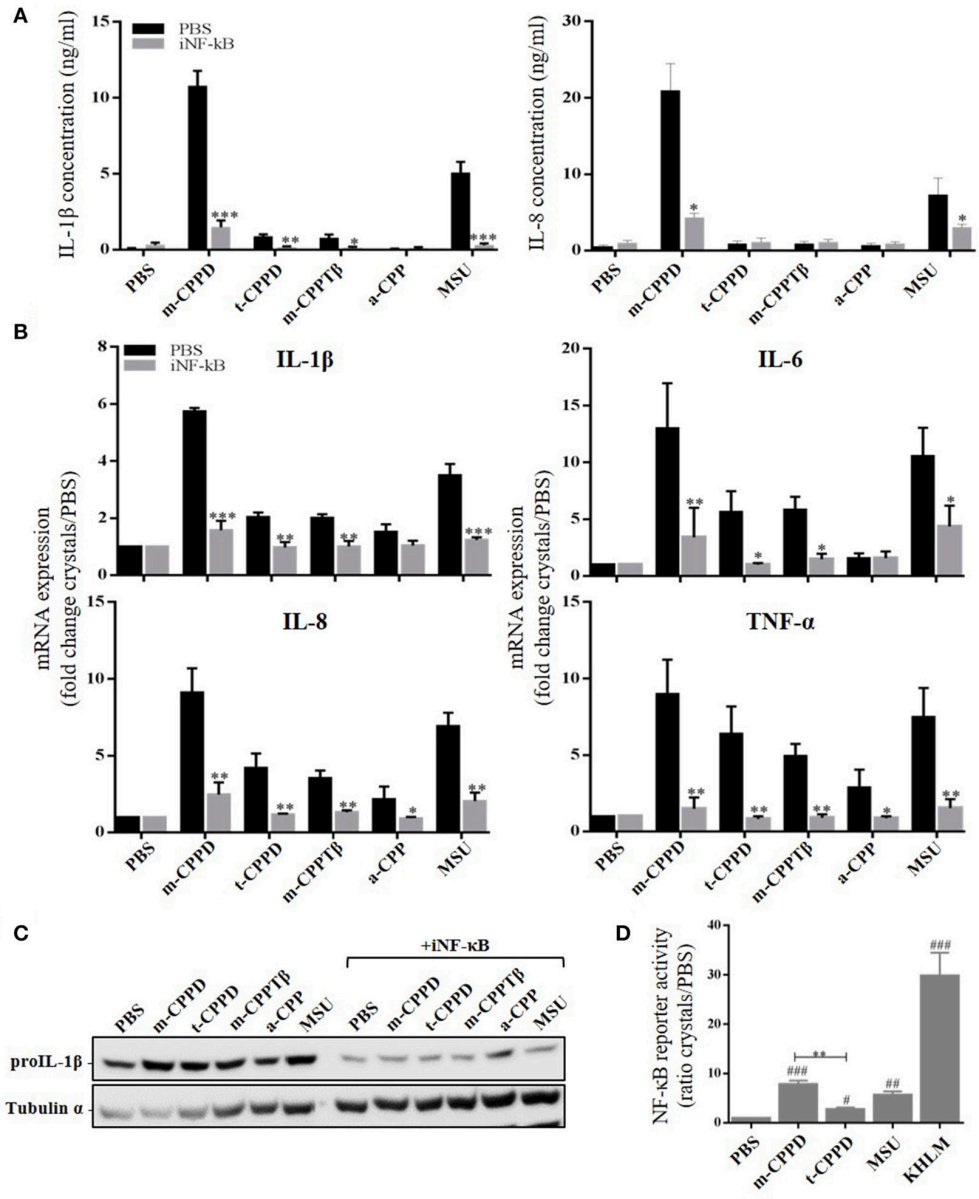
NF-κB regulates CPPD microcrystal-induced inflammation. Cells were primed the day before experiments. **(A–C)** 6 h after crystal stimulation of THP-1 cells, in the absence (PBS) or presence of NF-κB inhibitor (BAY-11-7085 [iNF-κB], 10 μM), supernatants were collected to quantify IL-1β (*n* = 5) and IL-8 (*n* = 3) concentrations **(A)**, and cells were lysed for analyzing IL-1β, IL-6, IL-8, and TNF-α gene expression by qPCR (*n* = 4) **(B)** or proIL-1β protein expression by western blot analysis (one experiment representative of three) **(C)**. Two-way ANOVA test with FDR correction: *p < 0.05; **p < 0.01; ***p < 0.001 (iNF-κB compared to PBS). **(D)** 8 h after crystal stimulation, supernatants of THP-1-Lucia cells were collected to determine the activity of secreted luciferase. Results are reported as relative NF-κB activity (*n* = 8); Kruskal-Wallis test with FDR correction between crystal and PBS (#) or between CPP crystals (*). #*p < 0.05; ##**p < 0.01; ###***p < 0.001. HKLM, heat-killed *Listeria monocytogenes*.

NF-κB is the main transcriptional factor leading to activation of multiple pro- and anti-inflammatory gene expression and therefore is strongly involved in microcrystal-induced inflammation. Thus, we wondered whether the inflammatory potential of different CPP crystals relied on their ability to activate this transcription factor. We first observed that primed THP-1 cell treatment with a pharmacological inhibitor of NF-κB (BAY-11-7085) greatly abrogated the production of IL-1β and IL-8 (Figure 6A) as well as the gene expression of inflammatory cytokines including IL-1β, IL-8, IL-6, and TNF-α (Figure 6B) induced by m-CPPD, t-CPPD, and m-CPPTβ microcrystals, as MSU crystals. Interestingly, BAY-11-7085 dose-dependently inhibited m-CPPD crystal-induced IL-1β production (Figure S1A, left).

**Figure 7:**
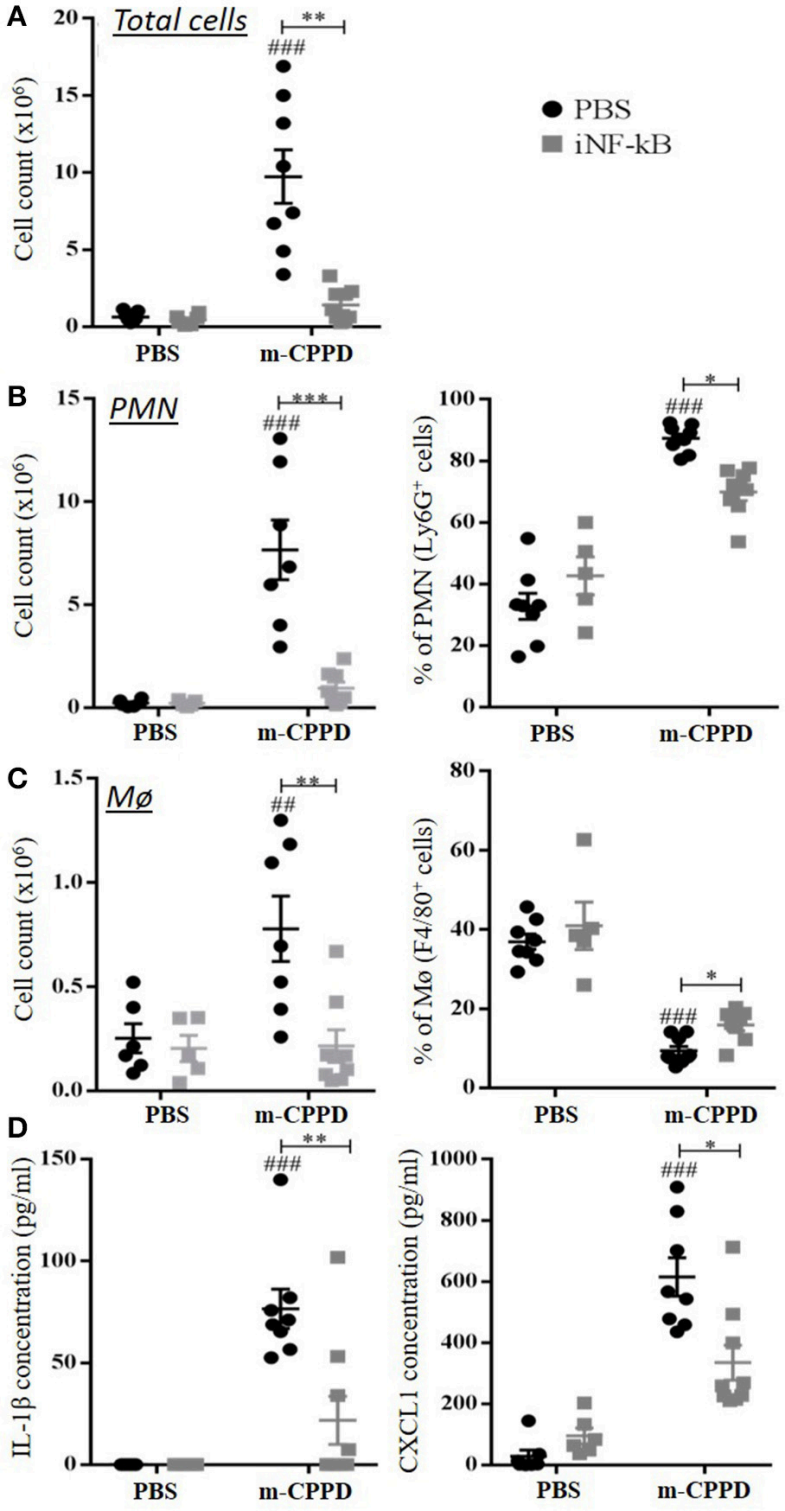
NF-κB regulates m-CPPD microcrystal-induced inflammation *in vivo*. Six hour after PBS or m-CPPD injection into the air pouches, in absence (PBS) or in presence of iNF-κB, the air pouch exudates were collected for analysis. **(A)** The number of total infiltrated cells was evaluated by cell counting with Trypan blue exclusion. **(B,C)** the absolute number (Left) and proportion (Right) of recruited neutrophils (PMN) and macrophages (Mø) among total cells was determined by flow cytometry after staining with anti-Ly6G-PE and F4/80-APC mAbs, respectively. **(D)** IL-1β and CXCL1 levels were quantified by ELISA. Kruskal-Wallis test with FDR correction between crystal and PBS (#) or between CPP crystals (*). ^#^*p < 0.05; ^##^**p < 0.01; ^###^***p < 0.001.

Then, we observed that the differential capacity of each phase of CPP crystals to induce proIL-1β formation was completely abolished by NF-κB inhibitor treatment (Figure 6C). This finding suggests that the decreased secretion of mature IL-1β was at least secondary to the inhibition of the first signal. To reveal NF-κB activation induced by crystals, we stimulated THP-1-Lucia cells containing a NF-κB-inducible luciferase reporter plasmid with the microcrystals. The ability to stimulate NF-κB was higher with m-CPPD than t-CPPD and MSU microcrystals (Figure 6D). Next, we validated the role of NF-κB *in vivo*. Indeed, PMN infiltration into the air pouch - defined by i) total cell count (Figure 7A), ii) absolute number and percentage of PMN or macrophages (Mø) (Figures 7B,C), and iii) production of IL-1β and CXCL1 (Figure 7D) - induced by m-CPPD microcrystals were greatly inhibited in mice pre-treated with the NF-κB inhibitor before crystal injection into the air pouch.

These results further demonstrate that the inflammatory response induced by CPP crystals is NF-κB-dependent, whatever the type of crystal, and that the inflammatory properties of m-CPPD, t-CPPD, and MSU crystals can be explained by their ability to differentially induce NF-κB signaling.

### CPPD crystals activate NF-κB via MAPK pathways

**Figure 8:**
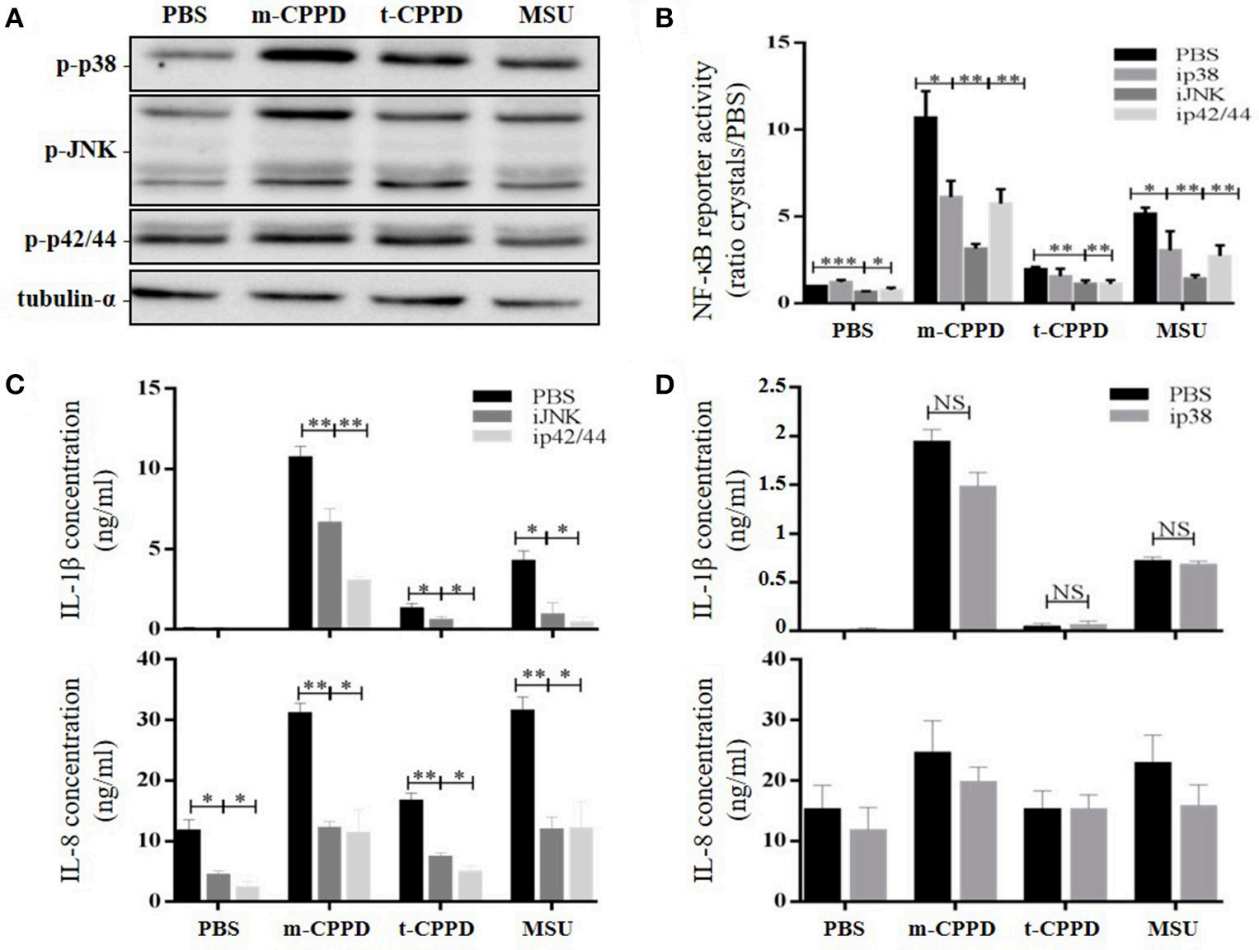
CPPD microcrystals activate NF-κB and induce inflammation via MAPK pathways. Cells were primed the day before experiments. **(A)** THP-1 cells were incubated for 30 min with crystals then lysed for immunoblot analysis of p38, p42/44, and JNK MAPK phosphorylation (one experiment representative of three). **(B–D)** Cells were stimulated with crystals in the absence (PBS) or presence of p38 (SB203580 (ip38), 10 μM), JNK (SP600125 (iJNK), 10 μM) or p42/44 (PD98059 (ip42/44), 100 μM) inhibitors. **(B)** THP-1-Lucia cell supernatants were collected at 8 h for determining luciferase activity (*n* = 4). HKLM, heat-killed *Listeria monocytogenes*. **(C,D)** THP-1 supernatants were collected at 6 h (**C**: ip42/44 and iJNK) or at 2 h (**D**: ip38) to quantify IL-1β and IL-8 levels by ELISA (*n* = 4). Multiple *t*-test with FDR correction PBS and MAPK inhibitors (*). *p < 0.05; **p < 0.01; ***p < 0.001.

NF-κB can be activated by several intracellular signaling pathways including TLR and MAPK pathways, both involved in CPP microcrystal-induced cell stimulation (13, 19). Thus, we evaluated - on primed THP-1 cells - whether the capacity of CPP crystals to activate NF-κB depended on p38, ERK1/2 and/or JNK MAPK pathways. As expected, we observed by immunoblot analysis that m- and t-CPPD crystals differentially stimulated the phosphorylation of these three MAPK pathways, with the effect of m-CPPD crystals again the most important (Figure 8A). Pharmacological inhibition of p38, JNK, or p42/44 (ERK1/2) MAPK decreased NF-κB activation induced by m-CPPD crystals (Figure 8B). Finally, IL-1β and IL-8 production induced by m-CPPD and t-CPPD were both substantially decreased by JNK and ERK1/2 MAPK inhibitors (Figure 8C) but were not modulated by the p38 MAPK inhibitor (Figure 8D). Both JNK and ERK1/2 MAPK inhibitors dose-dependently decreased m-CPPD crystal-induced IL-1β production (Figure S1A). Inhibition of JNK MAPK inhibition did not modify m-CPPD crystal-induced ERK and p38 MAPK activation. Similarly, ERK MAPK pathway inhibition did not alter m-CPPD crystal-induced JNK and p38 activation. In contrast, p38 MAPK inhibition increased JNK and ERK MAPK activation induced by m-CPPD crystals (Figure S1B). These results suggest that the inflammatory potential of CPP crystals depends on NF-κB activation induced by JNK and ERK1/2 MAPK pathways.

## Discussion

**Figure 9:**
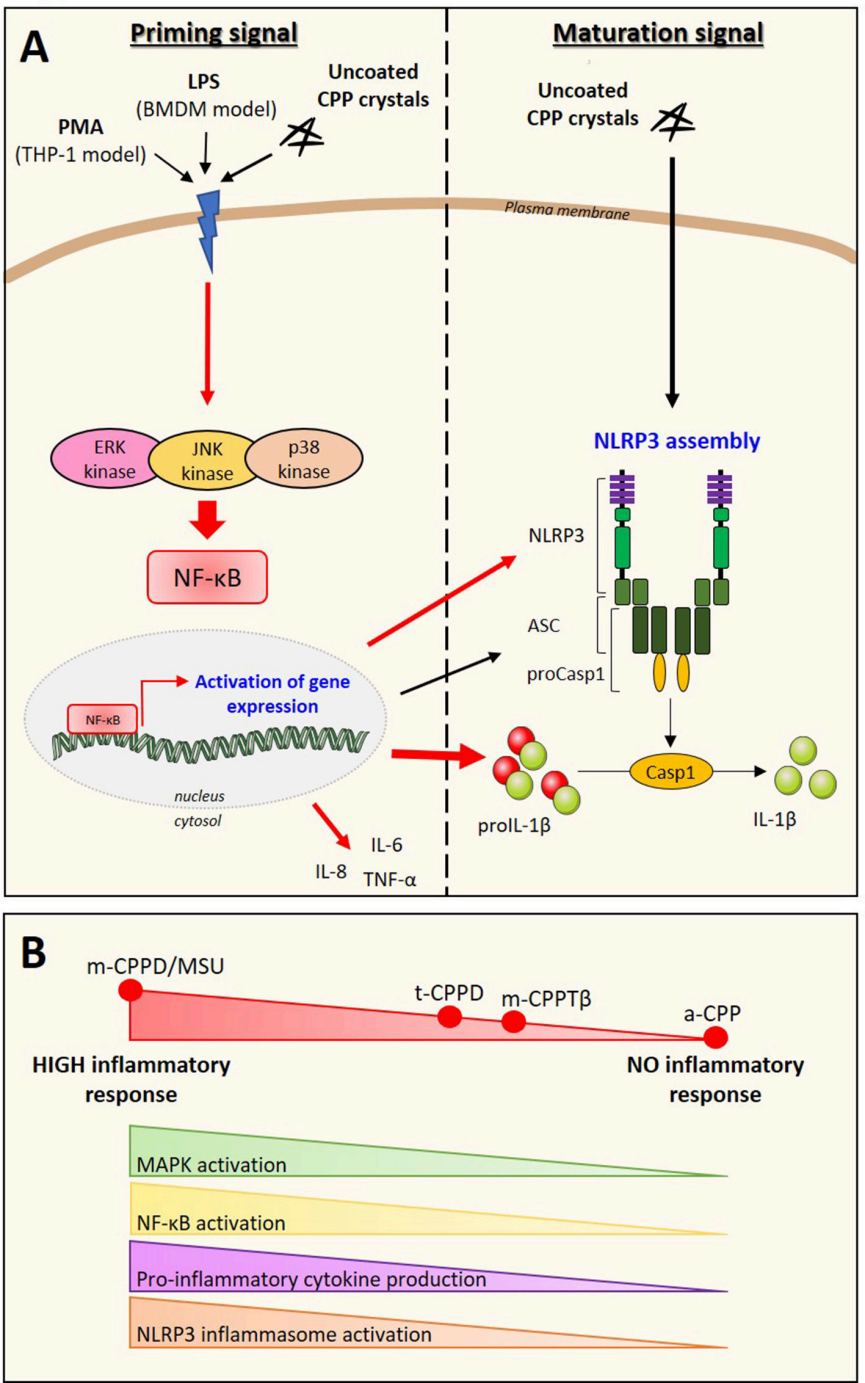
Schematic representation of the inflammatory potential of CPP crystals relying on both NF-κB (priming signal) and NLRP3 inflammasome (maturation signal) activation. **(A)** In our *in vitro* model of macrophages (THP-1 or BMDMs), uncoated CPP crystals act in synergy (red arrows) with PMA or LPS used for cell differentiation and activation. We showed that CPP crystals amplify NF-κB activation through a MAPK-dependent signaling pathways leading to an increased production of pro-inflammatory cytokines including TNF-α, IL-8, IL-6, and pro-IL-1β (**A**, left: priming signal). CPP crystals also enhance NLRP3 production and activation that induces ASC and pro-caspase-1 oligomerization (NLRP3 assembly) leading to caspase-1 maturation and subsequently cleavage of pro-IL-1β into active IL-1β (**A**, right: maturation signal). **(B)** CPP crystals possess different inflammatory properties with m-CPPD crystals being the most phlogistic ones followed by t-CPPD and m-CPPTβ, while a-CPP phase does not harbor inflammatory response. The inflammatory potential of each crystal depends on their ability to differentially activate the MAPK/NF-κB pathways and NLRP3 inflammasome complex. How CPP crystals differentially modulate NF-κB and NLRP3 remains unknown.

In this study, we report for the first time on the individual biologic inflammatory properties of four different CPP phases that have been recently fully described and characterized according to their large variability of morphology and size (4, 32). In contrast to most previous studies that assessed the cellular effects of mixed m-CPPD and t-CPPD crystals or poorly characterized CPPD crystals, we evaluated the biological properties of pure monophasic CPP particles. We observed a clear disparity effect of m-CPPD, t-CPPD, m-CPPTβ and a-CPP phases on inflammatory reactions. m-CPPD crystals were the most phlogistic crystals, followed by t-CPPD and m-CPPTβ, whereas a-CPP particles did not harbor inflammatory potential. The inflammation induced by m-CPPD crystals was more rapid and intense and lasted longer than that induced by t-CPPD and m-CPPTβ crystals. This disparity in response was observed *in vitro* and *in vivo* on the synthesis signal of pro-inflammatory cytokines (mediated by NF-κB and MAPK kinase activation) especially on the formation of immature pro-IL-1β as well as secretion of mature IL-1β, the central cytokine driving the microcrystal-induced arthritis (Figure 9). As for other particles, IL-1β production induced by each pure phase of CPP crystals was also dependent on NLRP3 inflammasome (17).

CPP crystal inflammatory properties depend on the potential induction of major inflammatory cytokines other than IL-1β, including IL-8, IL-6, and TNF-α. The role of IL-8 in CPPD and MSU crystal-related diseases is suggested in human diseases when IL-8 level is increased in serum and synovial fluid during gout and pseudo-gout flares, respectively (33, 34). Several *in vitro* and *in vivo* studies reveal the critical role of IL-8 in PMN recruitment and activation during MSU crystal-induced inflammation (35–38). Thus, blocking IL-8 activity by deleting its receptor CXCR2 or by anti-IL-8 antibody treatment abrogates neutrophil infiltration in mouse experimental models of MSU crystal-induced inflammation (36, 37). In contrast, until now, we lacked *in vivo* data of the role of IL-8 in CPPD crystal-related inflammation. Roch-Arveiller et al. previously showed that injection of m-CPPD crystals into the rat pleural cavity induced stronger and longer PMN recruitment than did injection of t-CPPD crystals (10). However, the authors did not evaluate the production of IL-1β and IL-8 or the involved intracellular signaling pathways. Moreover, the available *in vitro* data do not differentiate the individual effect of each type of CPP crystals on IL-8 production (38, 39). Here, we report that m-CPPD crystals, the most inflammatory CPP crystals, induced the highest level of IL-8 both *in vivo* and *in vitro*, which was correlated *in vivo* with IL-1β production and PMN recruitment at the site of crystal injection.

Therefore, our findings give some hints to explain the wide clinical spectrum associated with CPP crystal deposition. As suggested by Swan et al. (7), acute CPP crystal-associated inflammatory arthritis might be secondary to the presence of a large amount of CPP crystals with high inflammatory potential such as m-CPPD crystals, whereas asymptomatic CPPD might be associated with deposition of low-inflammatory CPP crystals such as t-CPPD or their potential precursors m-CPPTβ or a-CPP. This hypothesis needs to be confirmed by clinical studies assessing the precise phase of CPP compounds according to clinical phenotype.

In this study, we also confirmed that NF-κB was a major transcription factor involved in regulating IL-1β, IL-6, IL-8, and TNF-α gene expression induced by CPP crystals. Moreover, the inflammatory potential of each CPP phase tested to induce these genes depended on their ability to stimulate NF-κB activity via MAPK pathways, acting in synergy with PMA or LPS priming. Accordingly, previous groups showed that MSU crystal-induced IL-1β production was amplified by LPS or fatty acid priming (24, 40). In agreement with Liu et al. (38, 41) and Jaramillo et al. (28), we found that MSU and CPPD crystal-induced NF-κB activation depends on ERK1/2 and JNK MAPKs. Interestingly, we observed that the role of p38 MAPK in NF-κB activation differed for m-CPPD and t-CPPD crystals; the p38 MAPK inhibitor partially decreased m-CPPD crystal-induced NF-κB activation but had no effect on t-CPPD crystal-induced NF-κB activation. Also, p38 inhibition did not modify IL-1β production induced by m-, t-CPPD and MSU crystals while it decreased NF-κB activation induced by CPPD crystals. It might be secondary to off-target effects of the pharmacologic p38 inhibitors (42), especially on JNK and ERK MAPK activation (see Figure S1B). Nevertheless, these results suggested, first, the major role of JNK and ERK MAPK pathways, and second, the involvement of other transcription factors in CPP crystal-stimulated IL-1β production (43).

How CPPD crystals differentially activate intracellular signaling pathways remains unknown. In this study, the inflammatory potential of each studied CPP phase was not correlated with the value of SSA. Previous studies suggested that the inflammatory potential of microcrystals relied on their physicochemical characteristics including morphology, size, crystal-specific surface area and properties including crystal surface charge (or zeta potential), roughness and ability to adsorb proteins (6, 8, 10, 18, 21, 44). Most of these studies assessed basic calcium phosphate (BCP) and especially hydroxyapatite (HA) crystals. However, the role of these different crystal parameters varies depending on the study, and conflicting results are reported in the literature on the effect of crystal size, crystal-specific surface area and charge. Previously, we observed that the inflammatory potential of different BCP crystals including octacalcium phosphate, HA and carbonated-apatite crystals was correlated with specific surface area (8). In contrast, a few studies reported that inflammatory properties of HA crystals depended on their size and shape but not surface topography, surface charge or specific surface area. Thus, small and needle-shape HA crystals induced the strongest NLRP3 inflammasome activation and IL-1β production as well as TNF-α, IL-6, and IL-10 production, with limited responses triggered by larger crystals or similar-sized spherical and smooth particles (6, 44). These small-sized microcrystals were more efficiently internalized by monocytes or macrophages, and the amount of internalized crystals was correlated with inflammatory cytokine production (6, 45).

Besides crystal size, crystal morphology and surface charge also modulate cellular internalization capacity. Thus, more than 80% of positively charged particles were internalized after 1 h-incubation as compared with less than 5% of negatively charged particles (45). In our study, in contrast to BCP crystals, the inflammatory potential of the different CPP phases was mainly correlated with their structure (type of phase). Whether the inflammatory potential of CPP crystals is correlated with the amount of internalized crystals needs further study. Similarly, whether crystal aggregate formation modulates crystal phagocytosis and cellular responses deserves more investigation.

Crystal-induced cellular responses can be modulated by the ability of crystals to interact with the cell membrane. These interactions might occur through crystal surface charge, proteins adsorbed at the crystal surface, specific membrane receptors, channels or yet to be identified crystal receptors (17). The cellular effects of MSU and CPPD crystals, especially their inflammatory potential, are well known to be modulated by proteins adsorbed at the crystal surface (46–51). Thus, adsorption of apolipoprotein B on MSU crystals abrogates their inflammatory potential, whereas adsorption of immunoglobulin G (IgG) increases their inflammatory potential (46, 47). Similarly, t-CPPD crystals coated with serum proteins or IgG are more inflammatory than are uncoated t-CPPD crystals, whereas m-CPPD crystals coated with IgG are less inflammatory than are uncoated m-CPPD crystals (49, 50). Proteins adsorbed on MSU crystals change during inflammatory reaction, which suggests that adsorbed proteins are not strongly bound to the microcrystal surface (46, 47, 51). Whether the inflammatory potential of CPP crystal phases is secondary to their ability to adsorb different proteins remains unknown. A direct interaction between CPP crystals and cells is also hypothesized. Indeed, the simple atomic motif of the most developed face of t-CPPD could potentially destroy the double layer arrangement of phospholipid cell membranes (52). The differences between the crystalline structures of the CPP phases could then affect the ability to adsorb proteins. The organization of atoms and molecules on the surface of the different CPP crystals, and *a fortiori* on the a-CPP phase, presents a wide variety of configurations. The pyrophosphate molecule orientation and the number of calcium vary considerably between the fully hydrated basal surface of m-CPPTβ with no pyrophosphate molecules on the surface and the m-CPPD crystal faces with pyrophosphate molecules exposed almost parallel to this surface (53). Hence, the m-CPPD crystals could present enhanced properties of interaction with the environment, which could be responsible for its higher inflammatory property.

Because our experiments were performed with serum-free media, the CPP crystal inflammatory properties could essentially depend on their direct interaction with the plasma cell membrane. This hypothesis is further supported by the opposite effect reported with IgG coating on t-CPPD and m-CPPD crystals (49, 50). Moreover, direct interactions between crystals and the plasma cell membrane are strongly emphasized by the rapid activation of intracellular calcium mobilization and MAPK signaling pathways that occur within 1 min of crystal incubation (27, 54). Ng et al. nicely showed by atomic force microscopy that MSU crystals interacted with the cell membrane, which resulted in membrane cholesterol lipid sorting and intracellular recruitment of Syk kinase-dependent signaling and NLRP3 inflammasome activation (18). Recently, Hari et al. demonstrated that MSU crystals activated IL-1β production in the absence of phagocytosis; MSU crystals trapped at the bottom of the culture plate with a thin layer of epoxy were still able to activate macrophage inflammasome and IL-1β production (21).

In conclusion, our comparative study highlighted a differential inflammatory capacity of four different CPP phases. The three CPP crystals induced an NF-κB-dependent inflammatory response, each with a different level of pro-inflammatory cytokine expression and production, with m-CPPD the most powerful inflammatory crystal compared to t-CPPD and then m-CPPTβ. Also, we demonstrated for the first time that this inflammatory potential is explained by the ability of the crystals to differentially activate the MAPK-dependent NF-κB pathway. Finally, assessing how different CPP phases interact with the cell membrane would give insights into mechanisms that modulate the CPP crystal inflammatory potential and might explain the wide clinical phenotypes related to cartilage CPPD crystal deposition.

## Ethics statement

This study was carried out in accordance with the French national recommendations of Le comité national de réflexion d'éthique sur l'expérimentation animale. The protocol was approved by national ethical committee Le comité d'éthique en expérimentation animale Lariboisière/Villemin n°9 (APAFIS#6535).

## Author contributions

H-KE conceived the study. LC-G and H-KE contributed to its design and coordination, participated in data interpretation and co-wrote the manuscript. LC-G and FR performed the laboratory experiments and FR participated in data interpretation. MJ participated to laboratory experiments for revised manuscript. MG provided the nlrp3 knock-out mice. PG, CR, SS, and ChC synthesized CPP crystals, characterized their physico-chemical structure and contributed to writing the manuscript. CoC performed IL-6 quantification. H-KE, ChC, MC-S and FL secured funding. All authors read, participated in correcting and approved the final manuscript.

### Conflict of interest statement

The authors declare that the research was conducted in the absence of any commercial or financial relationships that could be construed as a potential conflict of interest.

## Supplementary material

Figure S1 Inhibitors of NF-κB and MAPK pathways dose-dependently decrease IL-1β production. THP-1 cells were primed the day before experiments. Then, cells were stimulated by m-CPPD crystals in the absence or presence of different doses of NF-κB [BAY-11-7085 (iNF-κB): 1–10 μM], JNK [SP600125 (iJNK): 0.1–10 μM], p42/44 [PD98059 (ip42/44): 1–100 μM] or p38 [SB203580 (ip38): 0.1–10 μM] inhibitors. **(A)** Cell supernatants were collected at 6 h of stimulation to quantify IL-1β by ELISA (*n* = 3). **(B)** Cell lysates were collected at 30 min of stimulation to assess MAPK activation by immunoblot (one experiment representative of three).
