# Supplementary figures and images for: Inflammatory Potential of Four Different Phases of Calcium Pyrophosphate Relies on NF-κB Activation and MAPK Pathways
Source: Front Immunol. 2018 Oct 9;9:2248. doi: 10.3389/fimmu.2018.02248 (PMC6189479; doi:10.3389/fimmu.2018.02248)

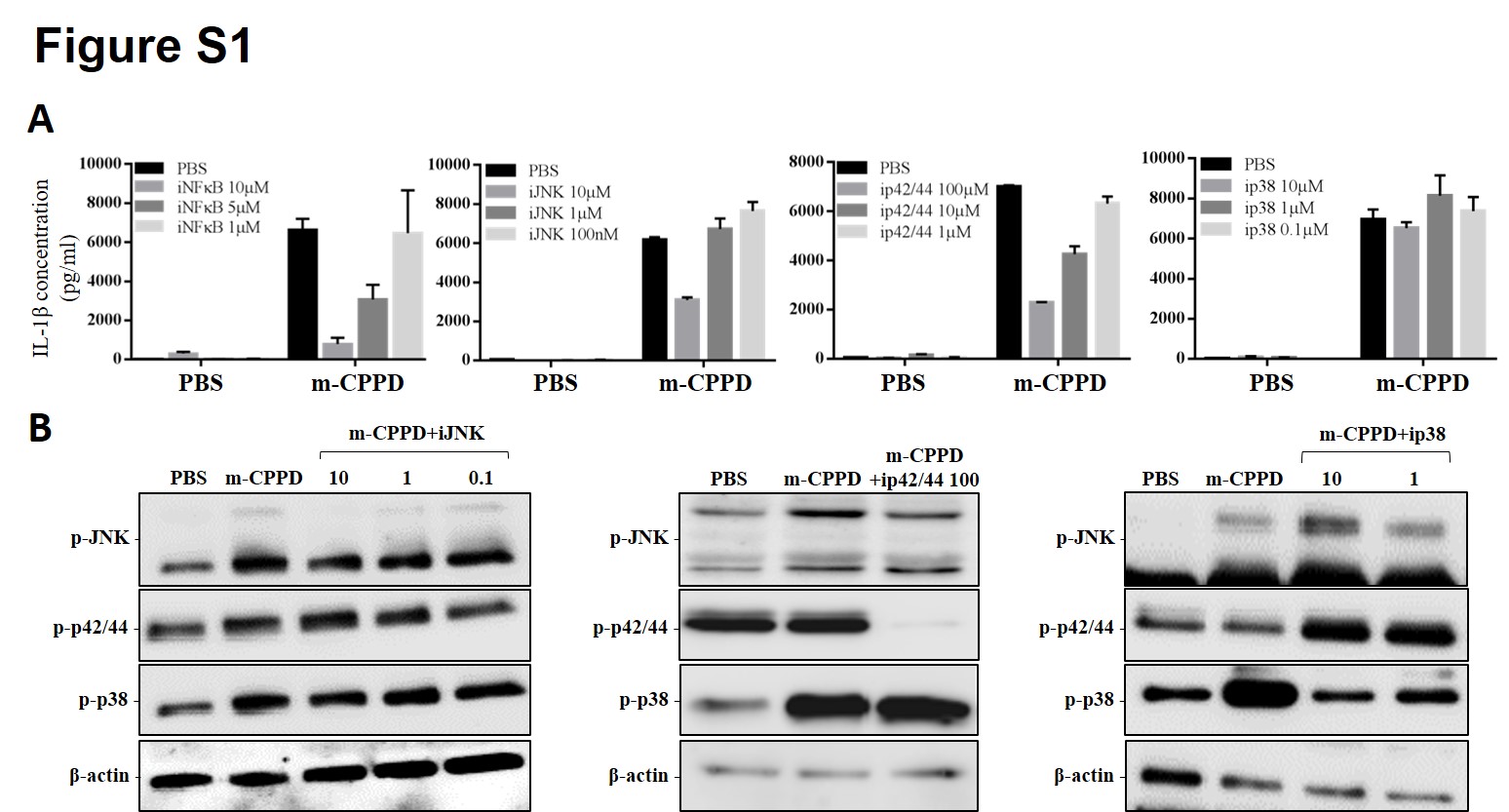

Supplement: Figure S1 — Inhibitors of NF-κB and MAPK pathways dose-dependently decrease IL-1β production. THP-1 cells were primed the day before experiments. Then, cells were stimulated by m-CPPD crystals in the absence or presence of different doses of NF-κB [BAY-11-7085 (iNF-κB): 1–10 μM], JNK [SP600125 (iJNK): 0.1–10 μM], p42/44 [PD98059 (ip42/44): 1–100 μM] or p38 [SB203580 (ip38): 0.1–10 μM] inhibitors. (A) Cell supernatants were collected at 6 h of stimulation to quantify IL-1β by ELISA (n = 3). (B) Cell lysates were collected at 30 min of stimulation to assess MAPK activation by immunoblot (one experiment representative of three). [file Image_1.JPEG]
